# Clinicopathological and Prognostic Characteristics of Esophageal Spindle Cell Squamous Cell Carcinoma: An Analysis of 43 Patients in a Single Center

**DOI:** 10.3389/fonc.2021.564270

**Published:** 2021-03-11

**Authors:** Peng Li, Yang Li, Chao Zhang, Yi-Hong Ling, Jie-Tian Jin, Jing-Ping Yun, Mu-Yan Cai, Rong-Zhen Luo

**Affiliations:** ^1^ State Key Laboratory of Oncology in South China, Collaborative Innovation Center for Cancer Medicine, Sun Yat-sen University Cancer Center, Guangzhou, China; ^2^ Department of Pathology, Sun Yat-sen University Cancer Center, Guangzhou, China; ^3^ Department of Pathology, The First Affiliated Hospital, Sun Yat-sen University, Guangzhou, China

**Keywords:** esophageal spindle cell squamous cell carcinoma, clinicopathological characteristics, prognosis, macroscopic type, perineural invasion, preoperative blood neutrophil to lymphocyte ratio, tumor size

## Abstract

**Objective:**

Esophageal spindle cell squamous cell carcinoma (ESCSCC) is a distinct subtype of esophageal carcinoma with unique morphologic and clinicopathologic features. This study aimed to characterize the clinicopathologic manifestations and postoperative prognostic factors of ESCSCC.

**Methods:**

In this study, 43 ESCSCC patients who underwent esophagectomy at Sun Yat-sen University Cancer Center between January 2001 and December 2014 were identified. 200 patients with conventional squamous cell carcinoma during the same period were sampled as a control. Hematoxylin and eosin-stained slides and available data were reviewed, and pertinent clinicopathologic features were retrospectively analyzed.

**Results:**

Among the ESCSCC patients, the median age was 60.5 years, with a male-to-female ratio of 2.58:1. The five-year disease-free survival and cancer-specific survival rates were 51.6 and 55.5%, respectively. In the univariate analysis, drinking abuse, tumor size, macroscopic type, perineural invasion, pT, preoperative blood white blood cell count, preoperative blood neutrophil count, and preoperative blood neutrophil to lymphocyte ratio were significantly correlated with the cancer-specific survival and disease-free survival of the ESCSCC patients. The multivariate analysis showed that macroscopic type, perineural invasion, and preoperative blood neutrophil to lymphocyte ratio were independent prognostic factors for cancer-specific survival; macroscopic type, perineural invasion, tumor size, and pT were independent prognostic factors for disease-free survival. Moreover, the combined prognostic model for cancer-specific survival (including macroscopic type, perineural invasion, and preoperative blood neutrophil to lymphocyte ratio), the combined prognostic model for disease-free survival (including macroscopic type, perineural invasion, and tumor size) significantly stratified patients according to risk (low, intermediate, and high) to predict cancer-specific survival, disease-free survival, respectively. In terms of esophageal conventional squamous cell carcinoma cohort, there was no significant difference in long-term outcome when compared with ESCSCC. Though five independent prognostic variables (macroscopic type, perineural invasion, preoperative blood neutrophil to lymphocyte ratio, tumor size, and pT) were indentified in ESCSCC, univariate analysis demonstrated that perineural invasion, preoperative blood neutrophil to lymphocyte ratio were correlated with esophageal conventional squamous cell carcinoma on cancer-specific survival; whereas only perineural invasion on disease-free survival.

**Conclusions:**

The proposed two new prognostic models might aid in risk stratification and personalized management for patients with esophageal spindle cell squamous cell carcinoma who received radical surgery.

## Introduction

Esophageal spindle cell squamous cell carcinoma (ESCSCC) is a rare subtype of esophageal squamous cell carcinoma, with unique morphology, histogenesis, and biological behavior. It accounts for 0.5–2.8% of all esophageal malignancies (1). Most ESCSCCs present as a gross intraluminal, polypoid mass. Histologically, ESCSCCs are composed of biphasic components of neoplastic squamous epithelium and spindle cells. The squamous part is always invasive and/or *in situ* squamous cell carcinoma, while the spindle cell element is usually malignant, which may show osseous, cartilaginous, or skeletal muscle differentiation (2, 3). Recent immunohistochemical, electron microscopic and genetic studies have provided support for the metaplastic concept, which states that the spindle cell component of ESCSCC exhibits various degrees of differentiation towards squamous cells and is a variant of poorly differentiated squamous cell carcinoma (4, 5). Therefore, ESCSCC was classified as subtype of esophageal squamous cell carcinoma in the current WHO classification (2019).

Radical esophagectomy with adequate lymph node dissection is the standard treatment for ESCSCC patients. Because of ESCSCC rarity, the long-term outcome of ESCSCC after radical surgery is controversial. Some investigators have suggested that ESCSCC treated with radical surgery has a comparatively better prognosis than that with esophageal conventional squamous cell carcinoma (6, 7). However, Sano et al. and Cavallin et al. have shown the opposite results (3, 8). During the past two decades, systemic adjuvant therapies, such as chemotherapy, radiotherapy, combination therapy, and targeted therapies, have been proposed to improve survival for ESCSCC patients with radical surgery (8–10). Minimizing the risk of overtreatment caused by non-selective use of these approaches, there is an urgent need to identify prognostic factors, especially for those with a high risk of tumor recurrence and poor prognosis. However, due to the controversy over ESCSCC’s long-term outcome and lack of widely accepted prognostic factors, there is no consensus on the clinical management and adjuvant treatment for ESCSCC patients who received radical surgery.

In the present study, we retrospectively analyzed a series of 43 consecutive ESCSCC patients with radical surgery in our institute, focusing on the clinicopathological characteristics and postoperative prognostic factors, then compared the results with a cohort of esophageal conventional squamous cell carcinoma. The aim was to propose new prognostic models that might aid in risk stratification and personalized therapy for patients with ESCSCC.

## Patients and Methods

### Patient Selection

The Institute Research Medical Ethics Committee of Sun Yat-sen University Cancer Center approved this study. We retrospectively collected a cohort of 43 ESCSCC patients who underwent radical esophagectomy between January 2001 and December 2014, from the pathological information system of the Department of Pathology of Sun Yat-sen University Cancer Center (Guangzhou, China). The cases were selected based on the following: (1) inclusion criteria: histologically confirmed primary esophageal spindle cell squamous cell carcinoma; complete follow-up data; (2) exclusion criteria: the percentage of spindle cell component was less than 10%; pTNM stage IV. Meanwhile, 200 patients with esophageal conventional squamous cell carcinoma during the same period were sampled. The inclusion criteria were shown as follows: histologically confirmed primary esophageal squamous cell carcinoma; complete follow-up data. The exclusion criterion was: pTNM stage IV.

For ESCSCC cohort, the clinicopathologic variables were obtained, including patient gender, age, smoking history, drinking history, tumor size, macroscopic type, tumor location, grade of conventional squamous cell carcinoma component, percentage of the spindle cell component, vascular invasion, perineural invasion, pT, pN, body mass index, level of serum alkaline phosphatase, level of serum lactic dehydrogenase, blood white blood cell count, blood neutrophil count, blood lymphocyte count, blood neutrophil to lymphocyte ratio, blood mononuclear cell count, blood eosinophil count, blood basophile count, hemoglobin, platelet count, disease-free survival time and cancer-specific survival time. According to the international criteria for the elderly, age was changed into a binary variable (≤65 year, or >65 year). Smoking abuse was defined as “consumption of tobacco for at least 6 months and at least one cigarette every three days”. Similarly, drinking abuse refers to “consumption of alcohol for at least 6 months and at least once per week”. With regard of body mass index, Chinese recommended standard (body mass index >24) was used for the criteria for overweight and obesity. According to the reference range of normal level, these blood variables involved in our study were classified as low, normal, or high. It is worth mentioning that the above blood cell-based markers were extracted from preoperative blood routine test. If there were multiple blood tests before the surgery, the one which was most close to surgery was adopted. The clinicopathological variables are detailed in **Table 1**. With regard to the cohort of esophageal conventional squamous cell carcinoma, only those variables indentified as independent prognostic factors in ESCSCC cohort were collected.

**Table 1:** Baseline characteristics of the patients with esophageal spindle cell squamous cell carcinoma.

Characteristics	Patients (N = 43)
Gender	
Male	31 (72.1)
Female	12 (27.9)
Age (years)	
≤65	32 (74.4)
>65	11 (25.6)
Smoking abuse	
No	19 (44.2)
Yes	24 (55.8)
Drinking abuse	
No	33 (76.7)
Yes	10 (23.3)
Tumor size (cm)	
≤6	34 (79.1)
>6	9 (20.9)
Macroscopic type	
Polypoid type	36 (83.7)
Infiltrative type	7 (16.3)
Tumor location	
Upper portion	3 (6.9)
Middle portion	26 (60.5)
Lower portion-esophagogastric junction	14 (32.6)
Grade of conventional squamous cell carcinoma component	
G1	2 (4.7)
G2	24 (55.8)
G3	17 (39.5)
Percentage of the spindle cell component (%)	
Low (≤85)	31 (72.1)
High (>85)	12 (27.9)
Vascular invasion	
Absent	31 (72.1)
Present	12 (27.9)
Perineural invasion	
Absent	33 (76.7)
Present	10 (23.3)
pT	
T1	16 (37.2)
T2	15 (34.9)
T3	12 (27.9)
pN	
N0	24 (55.8)
N1	11(25.6)
N2	7 (16.3)
N3	1 (2.3)
Body mass index	
Normal (≤24)	36 (83.7)
High (>24)	7 (16.3)
Preoperative level of serum alkaline phosphatase (U/L)	
Low (<45)	2 (4.7)
Normal (45–125)	40 (93.0)
High (>125)	1 (2.3)
Preoperative level of serum lactic dehydrogenase(U/L)	
Low (<120)	4 (9.3)
Normal (120–250)	39 (90.7)
Preoperative blood white blood cell count (10^9^/L)	
Normal (3.5–9.5)	30 (69.8)
High (>9.5)	13 (30.2)
Preoperative blood neutrophil count (10^9^/L)	
Normal (1.8–6.3)	30 (69.8)
High (> 6.3)	13 (30.2)
Preoperative blood lymphocyte count (10^9^/L)	
Low (<1.1)	3 (7.0)
Normal (1.1–3.2)	38 (88.4)
High (> 3.2)	2 (4.6)
Preoperative blood neutrophil to lymphocyte ratio	
Low (≤3.25)	26 (60.5)
High (>3.25)	17 (39.5)
Preoperative blood mononuclear cell count (10^9^/L)	
Normal (0.1–0.6)	26 (60.5)
High (>0.6)	17 (39.5)
Preoperative blood eosinophil count (10^9^/L)	
Normal (0.02–0.52)	40 (93.0)
High (>0.52)	3 (7.0)
Preoperative blood basophile count (10^9^/L)	
Normal (0–0.06)	31 (72.1)
High (>0.06)	12 (27.9)
Preoperative blood hemoglobin (g/L)	
Low (<130)	24 (55.8)
Normal (130–175)	19 (44.2)
Preoperative blood platelet count (10^9^/L)	
Normal (100–350)	31 (72.1)
High (>350)	12 (27.9)

### Follow-Up

The patients were followed up every three months for the first year and then every six months for the next two years and annually thereafter. Screening for recurrence was performed by a physical examination, endoscopy, esophageal barium examination, CT, and MRI. Cancer-specific survival refers to the period from the date of diagnosis until death from ESCSCC, esophageal conventional squamous cell carcinoma, respectively. Disease-free survival refers to the period from the date of diagnosis until the date of first recurrence, locoregional or systemic; all other events were censored.

### Pathological Evaluation

Tumor size was defined as the maximum diameter of the tumor. In terms of macroscopic type in ESCSCC, tumors which presented as a gross intraluminal and polypoid mass were classified as the polypoid type; while those with predominantly infiltrative growth pattern along esophageal wall were defined as the infiltrative type. In esophageal conventional squamous cell carcinoma, macroscopic appearance was classified as protruding type, ulcerative type, and diffusely infiltrative type.

All surgical specimens were processed according to standard pathological procedures. Two pathologists (PL and YL) independently reviewed all HE-stained slides of the primary tumors and regional lymph nodes without knowledge of the patient clinical parameters and the findings of the other reviewer. Any discrepancies were solved by simultaneous re-examination of the slides by both pathologists with a double-headed microscope. At least three slides per tumor were available for pathological evaluation, according to identical strict criteria.

The grade of conventional squamous cell carcinoma elements was determined based on the criteria proposed by the WHO Classification of Tumors of the Digestive System (2019); pT (tumor infiltration depth), and pN (lymph node status) were defined according to the 8th edition of the UICC/AJCC TNM (tumor-node-metastasis) Classification System (2017); vascular invasion was defined as the invasion of vessel walls by tumor cells and/or the existence of tumor emboli within an endothelium-lined space (11), and perineural invasion was defined as the presence of viable tumor cells in the perineural space (12).

### Statistical Analysis

A receiver operating characteristic (ROC) curve analysis was used to determine the optimum cutoff point for continuous variables (tumor size, percentage of the spindle cell component, blood neutrophil to lymphocyte ratio). The cumulative cancer-specific survival and disease-free survival rates were calculated by the Kaplan–Meier method, and differences between the patient groups were tested by the log-rank test in univariate analysis. A Cox proportional hazard model was employed to determine independent prognostic factors. All tests were two-sided, and *P* < 0.05 was considered to be statistically significant. IBM SPSS 20.0 statistical software was used to perform the statistical analyses.

## Results

### Patient Characteristics

A total of 43 patients with ESCSCC were included in the present study. The clinicopathological features for our ESCSCC cohort are presented in **Table 1**. Of the 43 patients, 31 (72.1%) were men, and 12 (27.9%) were women, with a male-to-female ratio of 2.58:1. The median age at the time of diagnosis was 60.5 years (range, 39.0 to 83.0 years). For the macroscopic type, 36 patients were defined as polypoid type (83.7%), and seven patients were defined as infiltrative type (16.3%). With regard to the pTNM stage, most patients were in early stages (stage I or II, 31 patients, 72.1%), whereas twelve patients (27.9%) were in stage III.

Radical esophagectomy with regional lymph node dissection was performed in all 43 ESCSCC patients. Postoperative therapy was given to five patients: four received radiotherapy, and one received concurrent chemoradiotherapy.

### Pathologic Features

**Figure 1:**
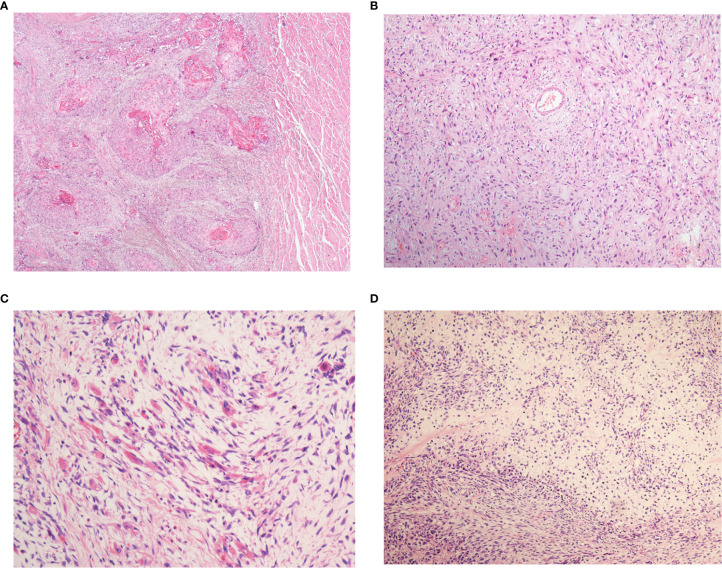
The histopathological patterns of esophageal spindle cell squamous cell carcinoma. All patients in the present study were composed of neoplastic squamous epithelium **(A)** and spindle-shaped sarcoma **(B)**. Definite mesenchymal differentiation, such as that in rhabdomyosarcoma **(C)**, chondrosarcoma **(D)**, is occasionally observed in the spindle cell components.

Microscopically, biphasic components of neoplastic squamous epithelium (invasive and/or *in situ* squamous cell carcinoma) and spindle-shaped sarcoma were observed in all 43 cases (**Figures 1A, B**). In addition, definite mesenchymal differentiation, including malignant peripheral nerve sheath tumor (one case), rhabdomyosarcoma/leiomyosarcoma (three cases, **Figure 1C**), or chondrosarcoma (one case, **Figure 1D**), was identified in the spindle cell components. The median percentage of spindle cell component was 65.5% (range, 10–95%). Regarding the depth of tumor invasion, sixteen tumors (37.2%) were superficial (T1), fifteen (34.9%) involved the muscular propria (T2), twelve (27.9%) involved the adventitia (T3). Lymph node metastasis was present in 19 of the patients (44.2%). Both the carcinomatous element and the spindle cell element have the potential for lymph node metastasis, with the predominance of a carcinomatous element. Vascular invasion and perineural invasion were detected in 12 patients (27.9%) and 10 patients (23.3%), respectively.

### Prognostic Factor Analysis

**Figure 2:**
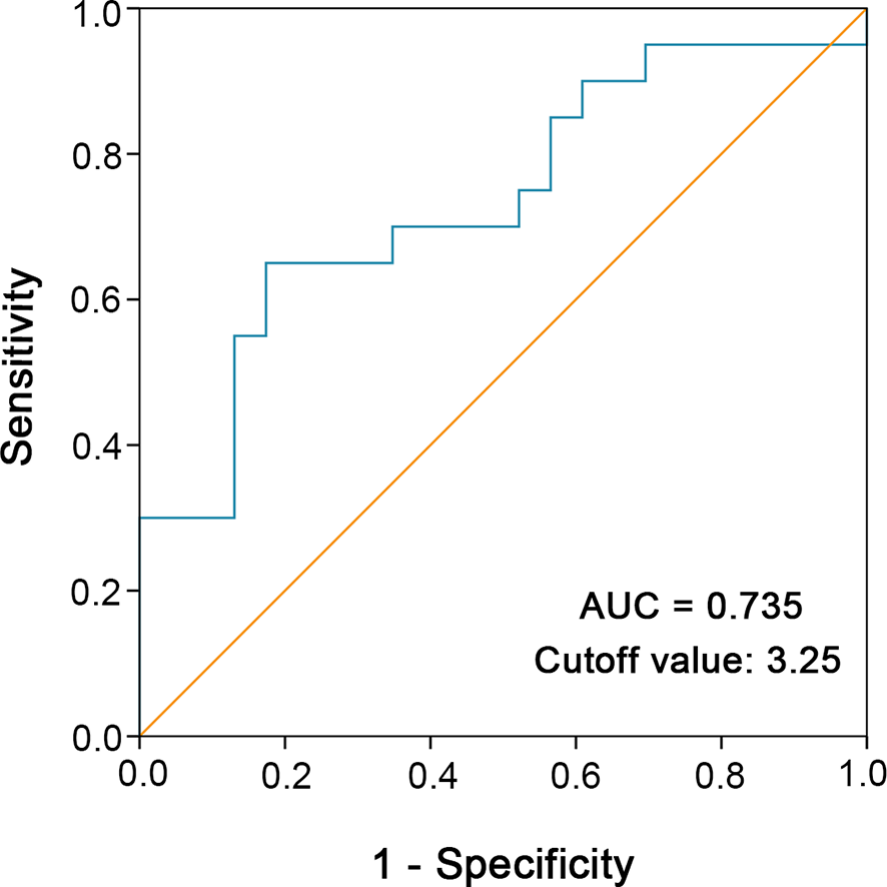
ROC curve analysis was performed to determine the optimal cutoff value of preoperative blood neutrophil to lymphocyte ratio. The sensitivity and specificity for the status of cancer-specific survival were plotted.

To determine the optimal cutoff value for continuous variables involved in our study (tumor size, percentage of the spindle cell component, blood neutrophil to lymphocyte ratio), we utilized the ROC curve to identify the cutoff point. For example, according to the ROC curve analysis, the cutoff value for preoperative blood neutrophil to lymphocyte ratio was 3.25 (**Figure 2**).

Until October 2018, the median follow-up time was 45.3 months, with a range of 2.8 to 146.5 months. At the end of the follow-up, 22 patients (22/43, 51.2%) experienced tumor recurrence, which presented as anastomotic or esophageal remnant recurrence, hematogenous spread, and lymph node metastasis. Hematogenous spread mostly occurred in the lung, thoracic vertebra, liver, and brain. Lymph node recurrence was present in mediastinal and abdominal aortic lymph nodes.

**Figure 3:**
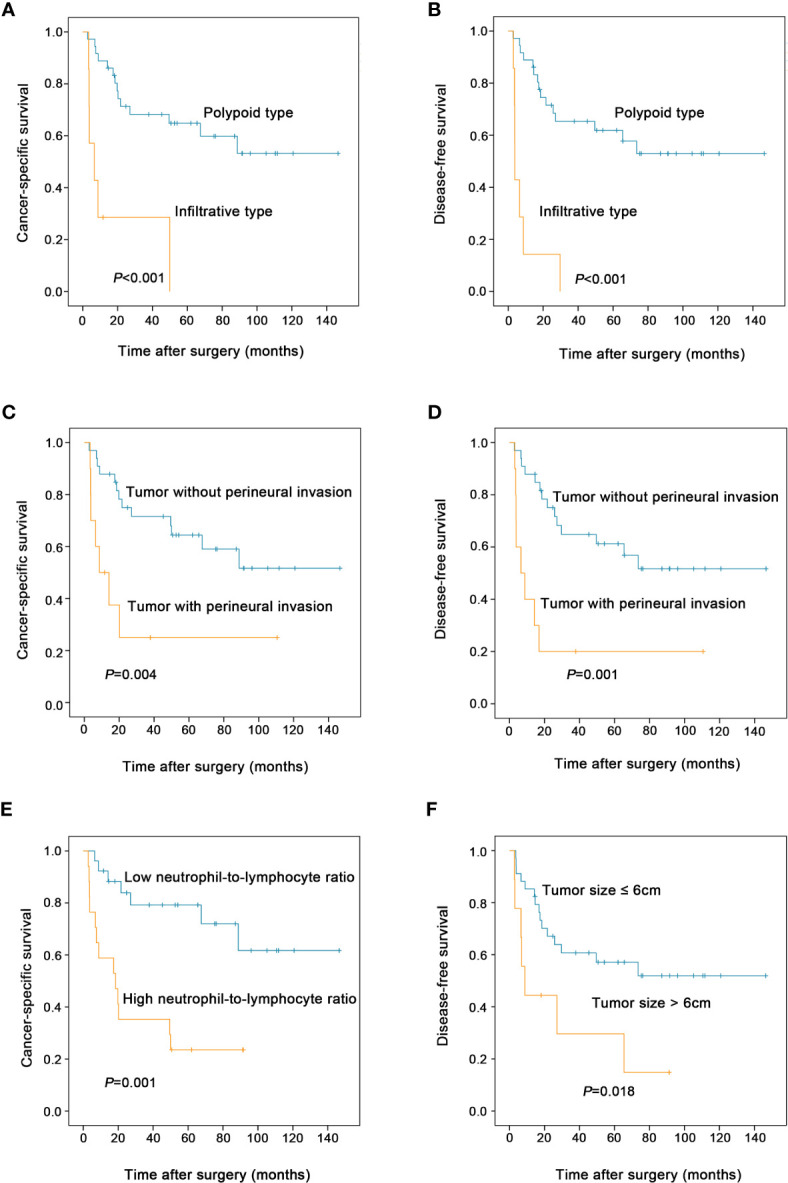
Prognostic factors affecting the postoperative survival of patients with esophageal spindle cell squamous cell carcinoma (log-rank test). Compared to polypoid type, infiltrative tumor type was associated with decreased cancer-specific survival **(A)** and disease-free survival **(B)** of patients. Tumor with perineural invasion had worse cancer-specific survival **(C)** and disease-free survival **(D)** than those without perineural invasion. High preoperative blood neutrophil to lymphocyte ratio was associated with decreased cancer-specific survival **(E)** in patients. Patients with tumor size >6cm had worse disease-free survival than those with tumor size ≤6cm **(F)**.

As shown in **Table 2**, the univariate analysis for cancer-specific survival showed that the variables significantly associated with ESCSCC included drinking abuse (*P =* 0.001), tumor size (*P =* 0.006), macroscopic type (*P* < 0.001, **Figure 3A**), perineural invasion (*P =* 0.004, **Figure 3C**), pT (*P =* 0.044), preoperative blood white blood cell count (*P=*0.011), preoperative blood neutrophil count (*P* = 0.001), preoperative blood neutrophil to lymphocyte ratio (*P =* 0.001, **Figure 3E**). With regard to disease-free survival, the significant prognostic factors in univariate analysis included: drinking abuse (*P* = 0.004), macroscopic type (*P* < 0.001, **Figure 3B**), grade of conventional squamous cell carcinoma component (*P* = 0.044), perineural invasion (*P* = 0.001, **Figure 3D**), tumour size (*P* = 0.018, **Figure 3F**), pT (*P* = 0.019), preoperative blood white blood cell count (*P* = 0.037), preoperative blood neutrophil count (*P* = 0.003), preoperative blood neutrophil to lymphocyte ratio (*P* = 0.002).

**Table 2:** Univariate analysis of clinicopathologic variables in patients with esophageal spindle cell squamous cell carcinoma for cancer-specific survival and disease-free survival (log-rank test).

Variables	Cases	Cancer-Specific Survival			Disease-Free Survival		
		Mean survival (months)	Median survival (months)	*P* value	Mean survival (months)	Median survival (months)	*P* value
Gender				0.062			0.129
Male	31	57. 7	50.0		54.7	49.5	
Female	12	114.7	NR		102.6	NR	
Age (years)				0.182			0.113
≤65	32	88.8	NR		85.3	73.5	
>65	11	35.0	27.1		29.3	27.1	
Smoking abuse							0.425
No	19	102.4	NR	0.125	88.9	NR	
Yes	24	55.7	50.0		55.4	49.5	
Drinking abuse				0.001			0.004
No	33	98.2	NR		90.8	NR	
Yes	10	29.7	8.7		29.4	8.7	
Tumor size (cm)				0.006			0.018
≤6	34	94.3	NR		87.4	NR	
>6	9	30.8	8.7		30.3	8.7	
Macroscopic type				<0.001			<0.001
Polypoid type	36	93.7	NR		90.8	NR	
Infiltrative type	7	18.0	6.5		8.4	3.8	
Tumor location				0.196			0.198
Upper portion	3	61.3	88.7		51.2	73.5	
Middle portion	26	95.6	NR		90.9	NR	
Lower portion- esophagogastric junction	14	34.9	21.6		30.1	21.6	
Grade of conventional squamous cell carcinoma component				0.114			0.044
G1	2	64.0	17.4		64.0	17.4	
G2	24	55.5	49.5		47.8	25.7	
G3	17	113.1	NR		113.4	NR	
Percentage of the spindle cell component(%)				0.533			0.844
Low (≤85)	31	74.1	NR		67.0	NR	
High (>85)	12	67.2	67.5		68.7	65.5	
Vascular invasion				0.240			0.119
Absent	31	89.3	88.7		85.8	NR	
Present	12	46.6	27.1		40.6	14.6	
Perineural invasion				0.004			0.001
Absent	33	93.6	NR		90.1	NR	
Present	10	34.5	8.70		28.1	6.5	
pT				0.044			0.019
T1	16	83.8	88.7		80.1	NR	
T2	15	81.8	49.5		81.8	49.5	
T3	12	40.9	8.7		34.5	6.9	
pN				0.158			0.078
N0	24	73.0	88.7		72.1	73.5	
N1	11	94.8	NR		85.3	65.5	
N2	7	47.9	20.2		36.4	6.9	
N3	1	8.7	8.7		8.7	8.7	
Body mass index				0.782			0.615
Low (≤24)	36	69.5	67.5		63.9	65.5	
High (>24)	7	89.0	NR		89.0	NR	
Preoperative level of serum alkaline phosphatase (U/L)				0.109			0.270
Low (<45)	2	28.0	6.5		28.0	6.5	
Normal (45–125)	40	87.2	88.7		81.7	73.5	
High (>125)	1	18.5	18.5		18.5	18.5	
Preoperative level of serum lactic dehydrogenase(U/L)				0.742			0.614
Low (<120)	4	66.3	67.5		68.8	65.5	
Normal (120–250)	39	82.2	88.7		76.2	49.5	
Preoperative blood white blood cell count (10^9^/L)				0.011			0.037
Normal (3.5–9.5)	30	96.6	NR		89.2	NR	
High (>9.5)	13	41.7	19.8		41.8	18.5	
Preoperative blood neutrophil count (10^9^/L)				0.001			0.003
Normal (1.8–6.3)	30	101.6	NR		93.9	NR	
High (>6.3)	13	23.1	19.8		22.8	16.9	
Preoperative blood lymphocyte count (10^9^/L)				0.808			0.938
Low (<1.1)	3	58.0	NR		39.1	29.7	
Normal (1.1–3.2)	38	80.3	88.7		78.3	65.5	
High (>3.2)	2	73.9	27.1		73.9	27.1	
Preoperative blood neutrophil to lymphocyte ratio				0.001			0.002
Low (≤3.25)	26	107.8	NR		100.0	NR	
High (>3.25)	17	34.0	18.5		32.3	16.9	
Preoperative blood mononuclear cell count (10^9^/L)				0.212			0.375
Normal (0.1–0.6)	26	73.2	NR		66.7	NR	
High (>0.6)	17	68.5	49.5		67.9	31.8	
Preoperative blood eosinophil count(10^9^/L)				0.974			0.837
Normal (0.02–0.52)	40	83.4	88.7		77.7	65.5	
High (>0.52)	3	37.0	NR		37.0	NR	
Preoperative blood basophile count (10^9^/L)				0.843			0.890
Normal (0–0.06)	31	85.9	NR		77.9	65.5	
High (>0.06)	12	66.7	88.7		64.5	73.5	
Preoperative blood haemoglobin (g/L)				0.655			0.966
Low (<130)	24	72.4	67.5		64.2	49.5	
Normal (130–175)	19	78.9	88.7		80.1	73.5	
Preoperative blood platelet count (10^9^/L)				0.266			0.168
Normal (100–350)	31	74.0	67.5		68.1	29.7	
High (>350)	12	83.9	NR		84.1	NR	

NR indicates not reached.

**Figure 4:**
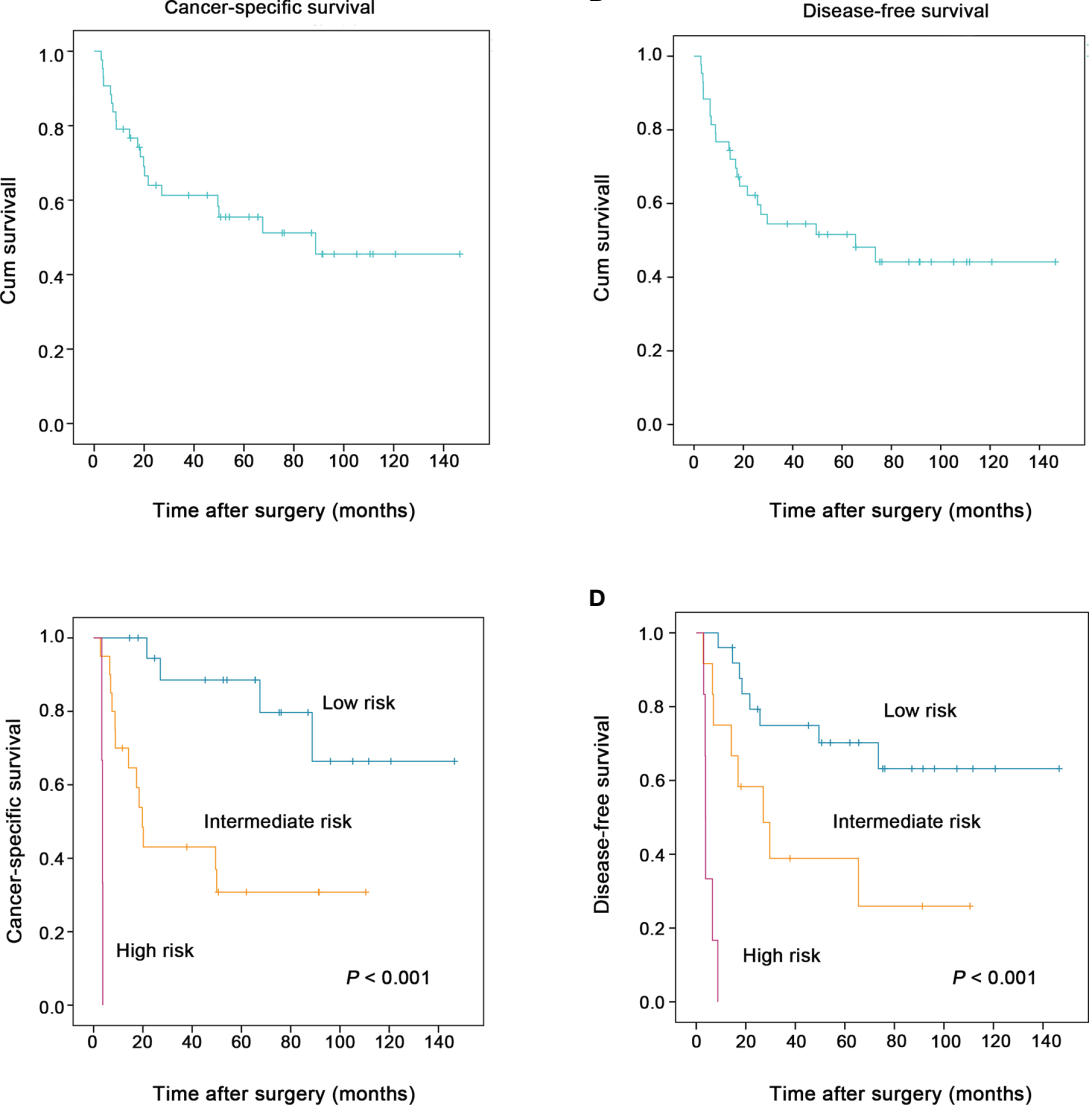
The two proposed prognostic models successfully stratified the risk of patients with esophageal spindle cell squamous cell carcinoma to predict survival (log-rank test). The overall cancer-specific survival and disease-free survival of patients in the present study are presented in **(A**, **B)**, respectively. The new combined model for cancer-specific survival (including macroscopic type, perineural invasion, preoperative blood neutrophil to lymphocyte ratio), another model for disease-free survival (including macroscopic type, perineural invasion, tumor size) clearly stratified patients into groups according to risk (low, intermediate and high) and was used to predict the cancer-specific survival **(C)**, the disease-free survival **(D)** of esophageal spindle cell squamous cell carcinoma patients, respectively.

Eventually, 20 patients (20/43, 46.5%) died of this tumor. The 1-, 3-, and 5-year cancer-specific survival rates were 79.1, 61.3, and 55.5% (**Figure 4A**), respectively. The 1-, 3-, and 5-year disease-free survival rates were 76.7, 54.5, and 51.6% (**Figure 4B**), respectively.

### Multivariate Cox Regression Analysis

To determine independent prognostic factors, we performed multivariate analysis for cancer-specific survival using a Cox proportional hazard model. Both all statistically significant variables in univariate analysis and the variable with p value in the range of 0.05–0.1 (gender) were included in the multivariate analysis. The results confirmed that macroscopic type (HR = 0.047, 95% CI 0.004–0.592, *P* = 0.018), perineural invasion (HR = 0.088, 95% CI 0.008–0.969, *P* = 0.047), and preoperative blood neutrophil to lymphocyte ratio (HR = 0.208, 95% CI 0.052–0.835, *P* = 0.027) were independent prognostic factors for cancer-specific survival (**Table 3**). However, macroscopic type (*P* = 0.006), perineural invasion (*P* = 0.005), tumor size (*P* = 0.013), and pT (*P* = 0.049) were found to be associated with disease-free survival independent of other clinicopathological parameters (**Table 4**).

**Table 3:** Cox multivariate analyses of prognostic factors on cancer-specific survival.

Variables	Hazard ratio	95% CI	*P* value
Gender (Male *v* Female)	1.499	0.283–7.944	0.634
Drinking abuse (No *v* Yes)	0.444	0.148–1.333	0.148
Tumor size (≤6 cm *v >*6* cm*)	0.319	0.091–1.116	0.074
Macroscopic type (Polypoid *v* Infiltrative type)	0.047	0.004–0.592	0.018
Perineural invasion (Absent *v* Present)	0.088	0.008–0.969	0.047
pT			0.265
pT (T1 *v* T3)	10.973	0.568–211.839	0.113
pT (T2 *v* T3)	6.092	0.334–111.035	0.222
Preoperative blood white blood cell count (10^9^/L) (Normal (3.5–9.5) *v* High(>9.5))	1.396	0.053–37.007	0.842
Preoperative blood neutrophil count (10^9^/L) (Normal (1.8–6.3) *v* High(> 6.3))	0.159	0.005–4.846	0.292
Preoperative blood neutrophil to lymphocyte ratio (Low *v* High)	0.208	0.052–0.835	0.027

CI, confidence interval.

**Table 4:** Cox multivariate analyses of prognostic factors on disease-free survival.

Variables	Hazard ratio	95% CI	*P* value
Drinking abuse (No *v* Yes)	0.445	0.111–1.785	0.253
Tumor size (≤6 cm *v >*6* cm*)	0.164	0.040–0.683	0.013
Macroscopic type (Polypoid *v* Infiltrative type)	0.003	0.000–0.189	0.006
Grade of conventional squamous cell carcinoma component G1 *v* G3 G2 *v* G3			0.565
	2.066	0.059–72.178	
	2.153	0.526–8.815	
Perineural invasion (Absent *v* Present)	0.023	0.002–0.322	0.005
pT			0.049
pT (T1 *v* T3)	136.727	2.489–7511.031	
pT (T2 *v* T3)	48.208	0.976–2380.761	
pN pN (N0 *v* N3*)* pN (N1 *v* N3*)* pN (N2 *v* N3*)*			0.095
	30.286	1.021–898.548	
	18.062	0.727–448.554	
	114.165	2.574–5063.553	
Preoperative blood white blood cell count (10^9^/L) (Normal (3.5–9.5) *v* High(>9.5))	1.116	0.016–78.718	0.960
Preoperative blood neutrophil count (10^9^/L) (Normal (1.8–6.3) *v* High(>6.3))	0.218	0.003–16.492	0.490
Preoperative blood neutrophil to lymphocyte ratio (Low *v* High)	0.254	0.056–1.147	0.075

CI, confidence interval.

### Two New Prognostic Models for Cancer-Specific Survival, Disease-Free Survival, Respectively

For cancer-specific survival, based on the three independent prognostic risk factors, macroscopic type, perineural invasion, and preoperative blood neutrophil to lymphocyte ratio, we built a new prognostic model to stratify the risk. The proposed model for cancer-specific survival confirmed that ESCSCC patients can be divided into a high-risk group (three risk factors), an intermediate-risk group (one or two risk factors), and a low-risk group (none of the above risk factors). Similarly, in terms of disease-free survival, we proposed a new prognostic model including macroscopic type, perineural invasion, and tumor size. The prognostic model for disease-free survival could classify ESCSCC patients into a high-risk group (two or three risk factors), an intermediate-risk group (one risk factor), and a low-risk group (none of the above risk factors). The two combined models significantly stratified risk (low, intermediate, and high) for cancer-specific survival, disease-free survival prediction, respectively (both *P* < 0.001, **Figures 4C, D**). Further analysis revealed that the 5-year disease-free survival rate was 70.2% in the low-risk group, 38.9% in the intermediate-risk group, and 0% in the high-risk group. The 5-year cancer-specific survival rate was 88.5% in the low-risk group, 30.8% in the intermediate-risk group, and 0% in the high-risk group.

### Comparison of Prognosis With Esophageal Conventional Squamous Cell Carcinoma

200 patients with conventional squamous cell carcinoma during the same period were sampled as a control. Five variables identified as independent prognostic factors in our cohort of ESCSC were collected, including macroscopic type, perineural invasion, preoperative blood neutrophil to lymphocyte ratio, tumor size, and pT. The clinicopathologic characteristics were detailed in **Supplemental Table 1**.

There were no significant difference between ESCSCC and esophageal conventional squamous cell carcinoma on the 5-year cancer-specific survival rate (55.5 *v* 42.0%, *P =* 0.384) and 5-year disease-free survival rate (51.6 *v* 41.5%, *P* = 0.588). Univariate analysis demonstrated that perineural invasion (*P* < 0.001), preoperative blood neutrophil to lymphocyte ratio (*P* = 0.021) were correlated with esophageal conventional squamous cell carcinoma on cancer-specific survival (**Supplemental Table 2**); whereas only perineural invasion on disease-free survival (*P* < 0.001, **Supplemental Table 3**). Two new prognostic models we proposed for ESCSCC failed to significantly stratified risk (low, intermediate, and high) on cancer-specific survival rate or disease-free survival rate in our cohort of esophageal conventional squamous cell carcinoma.

## Discussion

In the present study, based on a relatively large single-center cohort of 43 ESCSCC patients who underwent surgical treatment, we found that macroscopic type, perineural invasion, and preoperative blood neutrophil to lymphocyte ratio were independent prognostic factors for cancer-specific survival. However, macroscopic type, perineural invasion, tumor size, and pT were found to be associated with disease-free survival independent of other clinicopathological parameters. More importantly, two combined prognostic models we proposed can significantly stratify risk (low, intermediate, and high) to predict cancer-specific survival, disease-free survival, respectively.

Historically, ESCSCC is not a well-known entity. There are several synonyms, such as carcinosarcoma, sarcomatoid carcinoma, spindle cell carcinoma, metaplastic carcinoma, polypoid carcinoma, pseudosarcoma, squamous cell carcinoma with sarcomatous feature, squamous cell carcinoma with spindle cell features (3). These discrepancies in nomenclature reflect the limit knowledge of ESCSCC. In the WHO Classification of Tumors of the Digestive System (2019), ESCSCC is classified as the subtype of esophageal squamous cell carcinoma. Our findings support this classification. First, though it is companied by variable proportions of malignant spindle-shaped sarcoma element, there is no significant difference between ESCSCC and esophageal conventional squamous cell carcinoma in long-term outcome. Secondly, our research found several different prognostic factors only in ESCSCC, *e.g.* tumor size, macroscopic type, and pT. Thirdly, ESCSCC and esophageal conventional squamous cell carcinoma shared some common prognostic factors, such as perineural invasion, preoperative blood neutrophil to lymphocyte ratio. However, it is worth mentioning that in terms of preoperative blood neutrophil to lymphocyte ratio, the cutoff for esophageal conventional squamous cell carcinoma is 2.79 while it is 3.25 for ESCSCC. Lastly, two new prognostic models we proposed for ESCSCC failed to significantly stratified risk (low, intermediate, and high) in our cohort of esophageal conventional squamous cell carcinoma. Our findings demonstrated that the underlying molecular biological basis for ESCSCC might be at least in part different from that for esophageal conventional squamous cell carcinoma, supporting the notion that ESCSCC may be distinguished from esophageal conventional squamous cell carcinoma as a rare subtype.

Currently, the long-term clinical outcome of ESCSCC patients treated with radical surgery is controversial. Cavallin et al. found that the recurrence rate was 80%, leading to death within two years after surgery (8). The 5-year overall survival rate reported in other studies ranged from 44.8 to 61.9% (3, 6, 7, 13). Consistent with Sano et al. and Hashimoto et al.’s findings (3, 13), our study showed that the 5-year cancer-specific survival rate was 55.5%. Limited sample size, the quality of radical surgery, the percentage of patients in the early stage, and other prognostic factors might lead to these discrepancies in prognosis among different studies.

Our data showed that the percent of the spindle cell elements was not associated with cancer-specific survival and disease-free survival for ESCSCC patients who underwent radical surgery. These outcomes led us to speculate that both carcinomatous and spindle cell elements determine the malignant behavior of ESCSCCs. However, Natsugoe et al. found that cells in the sarcomatous and carcinomatous components were aneuploid and diploid, respectively, based on DNA flow cytometric analysis. They proposed the concept that the sarcomatous component in ESCSCC accounts for malignant behavior (14). Thus, which component in ESCSCC defines the degree of malignant behavior of this tumor is still controversial and needs further investigation.

In the current study, we paid special attention to the potential prognostic role of preoperative peripheral blood cell-based markers for ESCSCC. Currently, accumulating evidence has supported these blood cell-based markers as predictors of outcome after an operation and treatment response to neoadjuvant chemotherapy in various types of malignancies (15–20). In terms of our research, the elevation of preoperative blood neutrophil to lymphocyte ratio was independent predictor of poor cancer-specific survival for patients with ESCSCC who underwent curative surgical resection. Our observations might suggest a potential impact of cancer-associated inflammation on the progression and metastasis of ESCSCC. In general, the inflammatory microenvironment established by the tumor promotes its further malignant behavior by producing DNA damage and genomic instability, enhancing proliferation and survival, stimulating angiogenesis, favoring invasion and metastasis, and inducing an immunosuppressive environment (21, 22). Moreover, our analysis highlighted the role of neutrophils in ESCSCC malignant behavior, suggesting the potential application of future therapies targeting the tumor inflammatory microenvironment for ESCSCC patients.

pTNM stage is the best-established risk factor for important aspects affecting the prognosis of patients with esophageal cancer. This parameter, based on specific clinicopathological features and extent of disease, may have reached its limit in providing critical information in influencing patient prognosis and treatment strategies. Therefore, there is a need for new objective strategies that can effectively distinguish between patients with favorable and unfavorable outcomes. In our study, our data support the idea that macroscopic type, perineural invasion, preoperative blood neutrophil to lymphocyte ratio, and tumor size can effectively identify ESSC patients who may have aggressive clinical courses and adverse outcomes. Thus, macroscopic type, perineural invasion, preoperative blood neutrophil to lymphocyte ratio, and tumor size may become factors for predicting prognosis and render a more tailored treatment strategy in ESCSCC patients. Based on these interesting results, we propose two new prognostic models for cancer-specific survival, disease-free survival, respectively. The two proposed models may help to guide postoperative follow-up and individualized treatment.

Several limitations may affect the interpretation of this study due to the single-center retrospective design and the small sample size. However, given the rarity of the disease, larger prospective studies are difficult. In contrast, multi-center retrospective studies with a larger sample size should be encouraged. In addition, in our cohort of ESCSCC, five patients received postoperative therapy. Neoadjuvant treatment was not given in anyone patient with pTNM stage II or III. It was really disproportionately low compared to the current standard. Our cohort patients were retrospectively collected between January 2001 and December 2014. During this period, because of this tumor rarity, there was no consensus on the clinical management and adjuvant treatment for ESCSCC patients who received radical surgery.

## Data Availability Statement

The original contributions presented in the study are included in the article/**Supplementary Material**. Further inquiries can be directed to the corresponding authors. The authenticity of this article has been validated by uploading the key raw data onto the Research Data Deposit public platform (www.researchdata.org.cn), with the approval RDD number as RDDA2021001924.

## Ethics Statement

The studies involving human participants were reviewed and approved by The Institute Research Medical Ethics Committee of Sun Yat-sen University Cancer Center. Written informed consent for participation was not required for this study in accordance with the national legislation and the institutional requirements.

## Author Contributions

M-YC and R-ZL designed the research. PL reviewed HE-stained slides, analyzed the data, and wrote the manuscript. YL reviewed HE-stained slides. CZ performed follow-up of patients after surgery. Y-HL and J-TJ acquired clinicopathological data. J-PY reviewed the manuscript. All authors contributed to the article and approved the submitted version.

## Funding

This work was supported by grants from National Natural Science Foundation of China (81672407 and 81872001, to M-YC).

## Conclusions

We proposed two new prognostic models based on macroscopic type, perineural invasion, preoperative blood neutrophil to lymphocyte ratio, and tumor size that can effectively identify ESCSCC patients with a high risk of tumor recurrence and poor prognosis. This may aid in personalized management for patients with ESCSCC.

## Conflict of Interest

The authors declare that the research was conducted in the absence of any commercial or financial relationships that could be construed as a potential conflict of interest.
