# Supplementary material for: Clinicopathological and Prognostic Characteristics of Esophageal Spindle Cell Squamous Cell Carcinoma: An Analysis of 43 Patients in a Single Center
Source: Front Oncol. 2021 Mar 11;11:564270. doi: 10.3389/fonc.2021.564270 (PMC7991578; doi:10.3389/fonc.2021.564270)
Supplement: Supplementary file 1 [file Table_1.doc]

**Supplemental Table 1. Baseline Characteristics of the Patients with Oesophageal Conventional Squamous Cell Carcinoma**

| **Characteristics** | **Patients (N=200)** |
| --- | --- |
| Gender |  |
| Male | 158 (79.0) |
| Female | 42 (21.0) |
| Age (years) |  |
| ≤ 65 | 165 (82.5) |
| > 65 | 35 (17.5) |
| Tumour size (cm) |  |
| ≤ 4 | 132 (66.0) |
| > 4 | 68 (34.0) |
| Macroscopic type |  |
| protruding type | 91 (45.5) |
| ulcerative type | 107 (53.5) |
| diffusely infiltrative type | 2 (1.0) |
| Perineural invasion |  |
| Absent | 107 (53.5) |
| Present | 93 (46.5) |
| pT |  |
| T1 | 11(5.5) |
| T2 | 41 (20.5) |
| T3 | 148 (74.0) |
| pN |  |
| N0 | 89 (44.5) |
| N1 | 63(31.5) |
| N2 | 40 (20.0) |
| N3 | 8 (4.0) |
| Preoperative blood neutrophil to  lymphocyte ratio |  |
| Low (≤ 2.79) | 145 (72.5) |
| High(> 2.79) | 55 (27.5) |
