# Supplementary material for: Clinicopathological and Prognostic Characteristics of Esophageal Spindle Cell Squamous Cell Carcinoma: An Analysis of 43 Patients in a Single Center
Source: Front Oncol. 2021 Mar 11;11:564270. doi: 10.3389/fonc.2021.564270 (PMC7991578; doi:10.3389/fonc.2021.564270)
Supplement: Supplementary file 3 [file Table_3.doc]

**Supplemental Table 3. Univariate Analysis of Clinicopathologic Variables in Patients with Oesophageal Conventional Squamous Cell Carcinoma for Disease-Free Survival (log-rank test)**

| **Variables** | **Cases** | **Mean survival (months)** | **Median survival (months)** | ***P* value** |
| --- | --- | --- | --- | --- |
| Tumour size (cm) |  |  |  | 0.302 |
| ≤ 4 | 165 | 45.0 | 35.0 |  |
| > 4 | 35 | 38.6 | 23.0 |  |
| Macroscopic type |  |  |  | 0.479 |
| protruding type | 91 | 46.4 | 39.0 |  |
| ulcerative type | 107 | 40.5 | 27.0 |  |
| diffusely infiltrative type | 2 | 23.5 | 9.0 |  |
| Perineural invasion |  |  |  | <0.001 |
| Absent | 107 | 50.6 | NR |  |
| Present | 93 | 34.3 | 22.0 |  |
| pT |  |  |  | 0.151 |
| T1 | 11 | 49.5 | NR |  |
| T2 | 41 | 50.2 | NR |  |
| T3 | 148 | 40.6 | 27.0 |  |
| Preoperative blood neutrophil  to lymphocyte ratio |  |  |  | 0.077 |
| Low (≤ 2.79) | 145 | 45.8 | 40.0 |  |
| High(> 2.79) | 55 | 35.6 | 19.0 |  |

NR indicates not reached.
